# Nurturing positive mental health and wellbeing in educational settings – the PRICES model

**DOI:** 10.3389/fpubh.2023.1287532

**Published:** 2024-01-19

**Authors:** Tyralynn Frazier, Sebrina L. Doyle Fosco

**Affiliations:** ^1^The Center for Contemplative Sciences and Compassion-Based Ethics, Emory University, Atlanta, GA, United States; ^2^The Pennsylvania State University, University Park, Texas, PA, United States

**Keywords:** social and emotional learning, health promotion, wellbeing, student health, whole school, implementation, sustainable implementation, SEE learning

## Abstract

This article presents a comprehensive framework for the implementation of social and emotional learning (SEL) programs as a means of health promotion in educational settings, to positively impact student, school, and adult mental health and wellbeing across education. It emphasizes the profound impact of education on life trajectories and success definitions. Drawing inspiration from the Greek myth of Icarus and Maya Angelou’s poem “Still I Rise,” it explores contrasting notions of success and their consequences. Schools play a pivotal role in shaping students’ wellbeing across multiple dimensions and developmental stages. Because of this, holistic mental health and wellbeing promotion that takes a whole-school approach is critical. The paper introduces the PRICES framework (Preparation and Access, Restoration, Integration, Connection and Community, Educator Support, Strengths-Based Cultivation and Student Voice) as a comprehensive method for implementing SEL programs in educational systems. Each component of the PRICES framework is discussed in detail, emphasizing its role in fostering positive health promotion within schools. Examples of implementation plans that operationalize this model through a co-development process focusing on the Social, Emotional, and Ethical (SEE) Learning program are also presented. The PRICES model encourages a shift towards a more holistic approach to education, nurturing social and emotional development alongside academic achievement. By prioritizing wellbeing, fostering a sense of community, and integrating evidence based SEL interventions, schools can positively impact the mental health and overall flourishing of students and educators, contributing to thriving communities.

## 1 Introduction

How does education impact life trajectories through definitions of success? The Greek myth of Icarus serves as a timeless reminder of the perils associated with excessive and misguided ambition. Driven by his relentless pursuit of achievement, Icarus disregards his father’s wise counsel and pays the ultimate price, raising higher and higher towards the sun, only to fall. In contrast, Maya Angelou’s powerful poem “Still I Rise” celebrates resilience and perseverance in the face of adversity. “Leaving behind nights of terror and fear – I rise. Into a daybreak that’s wondrously clear – I rise. Bringing the gifts that my ancestors gave …”. Angelou’s vision of achievement draws strength from her ancestors and emphasizes the transformative power of community and connection in achieving success. In doing so, she continues to rise. Schools are catalysts for success, but it is how we define that success that shapes human health. In the story of Icarus, success is defined by a journey that ultimately harms the journeyer. With Angelou, the journey towards success is defined by an ongoing perseverance, aided by positive relationships that enable movement through challenges in a way that promotes connection, prosocial transformation, and sustained momentum. The journey is not harming the journeyer, it is transforming the journeyer towards health and resilience in the face of the inevitable challenges of the human condition.

What would it take for schools to be catalysts for this type of journey? This paper presents a framework for implementing social and emotional learning (SEL) programs that promotes whole-school wellness in a way that supports this type of transformational journey. Examples of this framework in action will be presented via implementation of the Social, Emotional, and Ethical (SEE) Learning program. In doing so, the aim is to reimagine school as a place where systemic processes support the sustained integration of whole-school SEL for maximum benefit in the communities those schools are serving.

## 2 Schools as catalysts for mental health promotion

### 2.1 The multiple dimensions of school

It has long been understood that educational institutions play a pivotal role in shaping the health and wellbeing of children. They provide a unique environment where children learn, grow, and flourish in multiple dimensions—academically, socially, emotionally, and behaviorally (1, 2). Beyond academic knowledge, schools define success and establish life trajectories for children based on these definitions. By the end of a child’s secondary educational period, referred to as high school in some countries, children are developing abstract ideas of their place in the world (3). During the creation of these ideas of what one’s life should be and who they will become, students are also navigating issues like bullying, academic and social pressures, and body image challenges (4). These challenges are compounded because the school years coincide with a dynamic period of physiological, neurobiological, and sociological changes (5, 6). Though this period has been characterized as one of strife and storm (7, 8), it is a time of dynamic becoming and school is at epicenter of this becoming.

### 2.2 Holistic mental health promotion

Schools are not merely centers for academic learning, they also serve as hubs for promoting health and wellbeing. Various health promotion practices have been integrated within schools, ranging from sex education to substance use prevention. In the last thirty years, social and emotional learning (SEL) has been added to this conversation on health (9–11) as a further catalyst for this healthy journey of becoming. While not explicitly framed as health promotion initiatives, these programs have consistently demonstrated positive impact on students’ social and emotional skills, academic achievement, behavior, and mental health outcomes (9). Schools clearly play a crucial social role beyond academics. By providing a platform for holistic growth, integrating health promotion practices, and fostering social and emotional skills, schools contribute significantly to the overall health and wellbeing of societies.

## 3 Social and emotional learning and the “work” of school

### 3.1 Two purposes

In asking “What is the work of school?” one is asking about the goal or purpose of complex institutions of education. Since the No Child Left Behind era, the focus appears to be almost entirely on academics, however, historically that has not always been the case. For example, the original intent for schooling in the United States (US) included the development of positive character traits such as honesty and compassion and promoting a “self-governing character” (12). Although full exploration is beyond the scope of this paper, the aim of this section is to illustrate that school is foundationally grounded in health promotion for two reasons, one implicit and one explicit. This intentional framing of the purpose of school is important because it shapes the fork in the road between the Angelou path and the Icarus path. The Angelou Path is defined as a life trajectory that emphasizes ongoing perseverance, resilience, and positive relationships as the keys to achieving success. On the Angelou Path, success is not harmful to the individual but rather serves to transform them, enabling health and resilience in the face of life’s challenges. This path promotes a prosocial and interconnected approach to personal and collective growth.

The Icarus Path is characterized by an excessive and misguided pursuit of achievement, often at the expense of oneself, disregarding the counsel of others and the potential consequences. In the Icarus Path, one strives for success through risky and unsustainable means. It can lead to a version of achievement, but ultimately harms the individual. It represents a journey where ambition and achievement take precedence over intrapersonal, prosocial development, and interconnected community development for sustained positive transformation. Being clear about what the work of school is enables a more direct focus on which path a school will be built to support.

### 3.2 Implicit in purpose – academic development and socialization

According to United Nations Educational, Scientific and Cultural Organization (UNESCO), the primary purpose of education, globally, is four-fold – 1. Academic and intellectual achievement, 2. political and civil purposes based on country or regional contexts, 3. Socialization, and 4. Economic growth and development (13). For example, the US National Educational Goals state purposes including providing quality education that enables all children to achieve their potential and promoting the development of socially productive democratic citizens (14). Explicit in this is the need to foster growth in knowledge and skills to develop students’ potential through academic learning. This learning is framed through the political orientation of democracy, and it necessitates support in navigating the social context – socialization, to support the entrance and the sustained engagement with an economic system (i.e., getting a job and achieving sustained financial security). This means that while academic learning is an explicit goal, embedded implicitly within this is developing the ability to navigate social contexts. Achieving these goals begins with academic learning to build knowledge and skills. This learning happens in a cultural context, and the goal of this contextualized learning journey is to increase economic opportunities. All these pieces involve processes of socialization, which, through a developmental perspective, involves the development of social skills within a social institution. How that socialization is framed leads to the explicit purpose of education (15).

### 3.3 Explicit in purpose – student health and wellbeing during development

Schools have long been acknowledged as a location to support health and wellbeing. During the school-aged period (ages 5–18), children are experiencing rapid, sequential changes in physical, linguistic, emotional, and social (16, 17) aspects of themselves. These types of changes occur throughout a lifetime, but this period is when children experience critical windows of malleability. For example, during early elementary years (ages 5–8), children students learn basic social skills that are foundational for navigating relationships, understanding values, building self-efficacy around responsibilities, and feeling safe to explore new ideas and activities. In the school context, the teacher-child relationship is of primary focus during this social and emotional development period. Teachers and primary caregivers play a critical role in supporting a child’s sense of security and competency through the dynamics of the attachment relationship. These relationships support academic learning (18–20) and long-term wellbeing outcomes.

During the later elementary years (ages 9–11) there is a shift toward greater emphasis on peer and friend groups, a push towards greater independence in decision-making, and a need to build competencies that are self-driven (21, 22). Developmentally, this period is focused on peer relationships and opportunities to support cooperation, empathy cultivation, conflict resolution and healthy collaboration, while also supporting the emergence of a self-concept that is strength-based and grounded in compassion.

During middle and secondary school years (ages 12–18), peer relationships continue to be most influential as social bonds through shared interests and values are emerging. Later, more intimate relationships start to emerge. Opportunities for navigating high-risk situations, making ethical decisions, and choosing healthy actions independently is very important (23–26). These navigations – relationships, behavioral choices, and academic successes – influence the consolidation of the self-identity that is emerging. By the end of this period, youth are developing a clearer sense of who they are in the world, what they want to do, and how they want to orient their values as they do it. It is also a critical period where the school environment, as a whole, needs to be intentional about providing supports that promote the positive navigation of relationships, stress and coping skills, and personal self-concepts that promote positive health. Students also need opportunities for learning how to navigate normal human challenges in relationships, identities, and professions. During this period, nurturing adults are crucial for guidance to support healthy development.

While the work of school includes academic achievement, the goals of school are indelibly linked to biological processes that orient the student towards self-engagement (intrapersonal), relationship engagement (interpersonal), and interactions with the systems that hold and shape their becoming. This means that school systems have a critical imperative to be intentional about how intrapersonal, interpersonal (27–29), and systems-level capacities are shaped to support positive development. This intentionality lays the foundation for positive health promotion (30–34).

Through these developmental spaces, there are clear opportunities for school to determine which path of support – the Angelou Path vs. the Icarus Path – they are fostering. With concerted effort, schools can be or become cultivators of the Angelou Path, but it takes intentionality. The PRICES framework presented below is an example of how schools and school districts can intentionally engage the Angelou Path to support a type of systemic development the promotes the long-term wellbeing of students and adults.

## 4 The PRICES framework as an implementation vehicle for educational health promotion

In this section, the PRICES model is used to illustrate an evidence-based and comprehensive method of SEL as a form of health and wellbeing promotion across educational systems (35). The significance of the PRICES framework lies in its identification of pivotal areas within complex dynamic systems, such as schools. Health promotion programs often are brought into these complex systems without attention to building a knowledge base about the program across the system. Further, a lack of attention to opportunities for practice and development across the system can lead to siloing of the program rather than the concepts being integrated for more universal impact. Finally, there can be a perception of hypocrisy other issues of equity in the system have not been addressed. The strategic attention to these domains within a comprehensive school-wide strategy engenders an environment conducive to the lasting and comprehensive implementation of health and wellbeing promotion efforts (35, 36).

This framework serves as a guiding tool for educational institutions, allowing them to adopt a system-wide and holistic approach to implementing social and emotional learning, effectively integrating proven methods from universal health promotion programs in educational settings and whole-school social and emotional program implementation for both high impact and sustainability (35). This approach not only facilitates the systemic amalgamation of these efforts but also ensures the sustainment of such initiatives within the educational landscape. This section describes the constituent elements of the PRICES framework and their role in fostering positive health promotion.

### 4.1 The PRICES framework

P – Preparation and access

R – Restoration

I – Integration

C – Connection and community

E – Educator support

S – Strengths-based cultivation and student voice

## 5 Application of the PRICES – whole school implementation

To illustrate the PRICES model, in the following section, after a description of the domain, we provide detailed examples of how schools navigated these domains during the implementation planning process for the SEE Learning program. We also provide key take-aways from our work with these schools.

SEE Learning is an innovative K-12 education program that provides a universal and science-based approach to bringing the ethical development of the whole child into education. The program modules explore dimensions of awareness, compassion and engagement within individual, social, and systems-level domains. SEE Learning expands on the field of SEL by drawing from new developments in educational practice and scientific research. Key additional components include attention and awareness training, compassion for self and others, trauma and resilience-informed care, systems thinking, and ethical discernment.

The goal for this research is to shed light on the process of establishing long-lasting, sustainable implementation of a program like SEE Learning that can positively impact health and wellbeing. The methods described below were employed separately in two school networks: one in a public school district and the other in a private school network. Key members of the research team and school leadership from both schools participated in developing the implementation plan for SEE Learning using an adapted version of the Community-Engaged Design Thinking (CEDT) Process, which has been used in community-based healthcare research (37, 38). The method involved a structured co-creation workshop to brainstorm, design, and develop the implementation plan based on the whole school domains.

Schools around the world implementing SEE Learning are guided though a whole-school implementation process that centers on crucial domains of the PRICES model. Initially, schools commit to adopting a comprehensive, holistic approach to recognize and address sustainable implementation needs and opportunities throughout the school. They then engage in a co-design process to create a sustainability plan based on the initial assessment. Finally, they put this plan into action to gauge the program’s long-term success and identify areas for refinement.

This process begins with guided planning sessions that includes educational leaders, teacher leaders and other staff, students, and parents in leadership roles. The objective is to pinpoint opportunities for integrating SEE Learning across various school domains defined in the present model. These domains serve as the foundation for sustainable change. SEE Learning uses this process to emphasize the importance of not only considering all domains but also gradually expanding efforts across each one.

**Figure 1:**
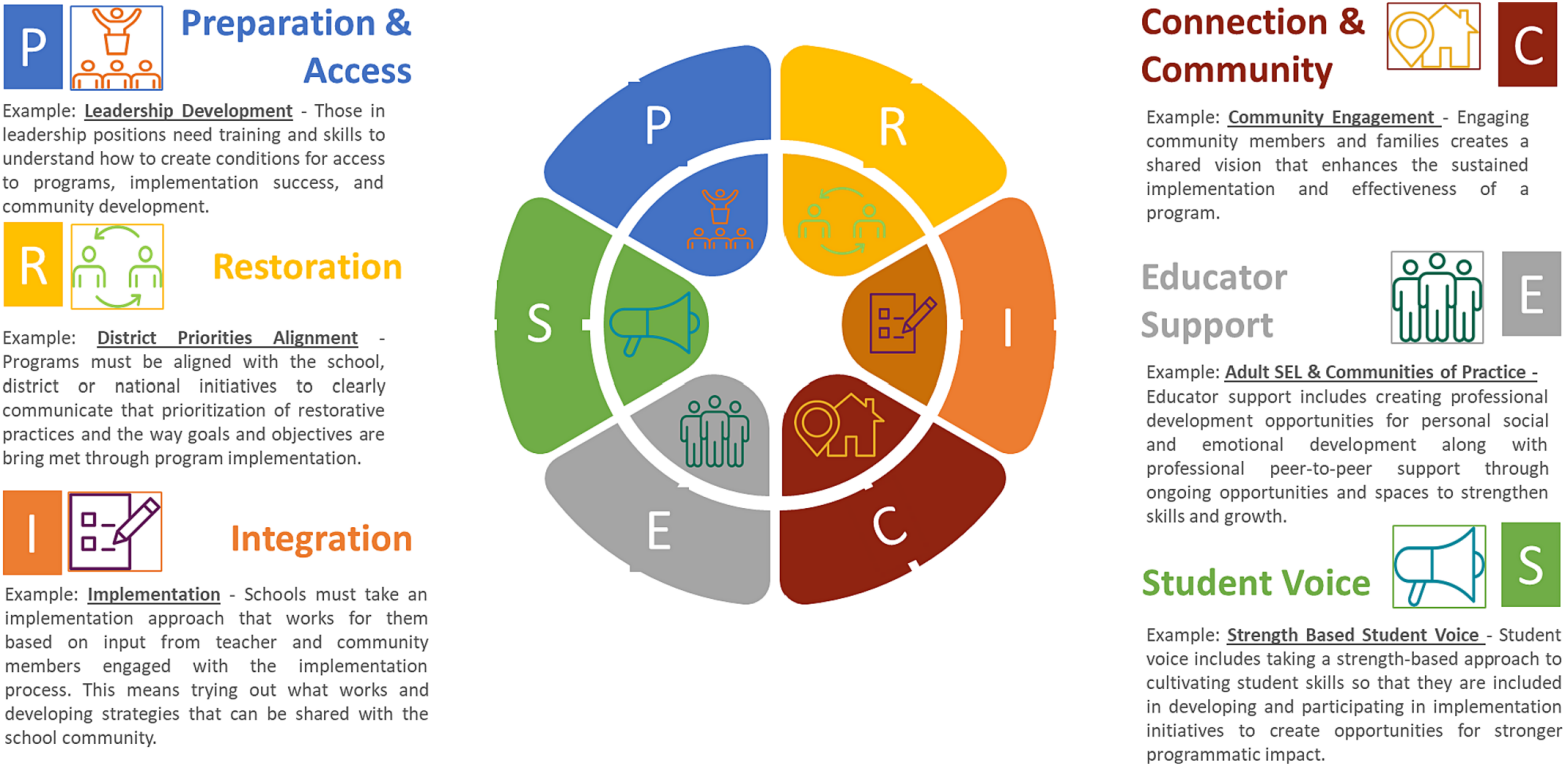
The PRICES model with examples of use. This figure illustrates the domains within the PRICES model and provides examples within each descriptive box of the ways each of these domains can be practically applied to any given school context.

For this research, the development process spanned two two-hour sessions and resulted in the identification of key actions that could be applied within the implementation framework. The domains are described in Figure 1. These plans were shared with teachers and staff across the entire school communities for their input and revision. The findings from this work are summarized as key actions identified across both networks as practical approaches to implementing after the description of each domain.

### 5.1 Preparation and access

#### 5.1.1 Preparation and access

Access to resources that cater to students’ material and psychological needs is a foundational requirement in schools. Achieving this involves recognizing, understanding, and nurturing students’ potential for academic and personal growth. This understanding draws from the contributions of various educational thinkers, such as John Dewey (39), who emphasized the pivotal role of students’ experiences, social interactions, and emotional wellbeing in their educational journey. Additionally, Bronfenbrenner’s ecological systems theory (40) sheds light on how student development is shaped by their immediate surroundings (microsystem) and broader cultural and societal factors (macrosystem). Effectively addressing both psychological and material aspects within these systems is essential for providing impactful education. Vygotsky’s sociocultural theory (41) further highlights the significance of social interaction and scaffolding, where students benefit from guidance and support from both peers and adults. This necessitates creating environments that facilitate this scaffolding. School leaders play a pivotal role in shaping the educational landscape by serving as gatekeepers to opportunities for both educators, staff, and students.

Leadership development is crucial in preparing the environment for this type of support. Educational leaders are instrumental in providing access to these resources. As educational institutions embrace health and wellbeing promotion initiatives, it is imperative for leaders to possess fundamental knowledge about supporting the professional development of educators and effective SEL implementation within the educational environment. Leaders should also understand how to create opportunities for accessing programs, practicing skills within these programs, and providing ongoing training and support for new educators entering the system. These types of opportunities include creating spaces that are nurturing and allow for dedicated time to foster social and emotional growth. This also includes creating space for the teachers to have time to participate in professional development and supportive peer networks for ongoing development.

To achieve this, educational leaders need development to support their own social and emotional competence and wellbeing (42, 43). This enables them to provide invaluable support for the professional growth of educators, who play a crucial role in recognizing and nurturing students’ capacities for academic and personal growth (43, 44). Leaders can mentor and guide their staff, helping them to develop and implement initiatives confidently. They can also create spaces and contexts for staff, which is crucial for the sustainable success of any endeavor within the school environment, directly impacting students’ access to resources that foster their readiness for success. Leaders can also signal support for such practices by extending the use of these skills and practices beyond the classroom and into administrative offices and meetings, fostering a shared language and understanding. This approach creates a collaborative environment that signals commitment to effective communication and emphasizes the value of programs that support social and emotional development.

##### 5.1.1.1 Example of preparation and access via SEE learning – leadership development

Example: The school teams expressed that leaders play a pivotal role as implementors by grounding the staff in the “why” of any program for both the teachers and the staff. In our work with schools on determining actions that could be taken to support this type of leadership development, the following are the top ten initiatives that schools said they could do in implementing the SEE Learning program.

Key takeaways – leadership

1. Develop a comprehensive leadership support plan based on what has been learned about what is needed to implementation of SEE Learning and other SEL programs.
2. Engage school leaders in SEE Learning facilitator training to develop their understanding of the program and how best to support their community.
3. Integrate SEE Learning practices into faculty and department meetings to enhance the implementation.
4. Create a dedicated leadership position to support SEE Learning implementation, and SEL implementation in general, at the district level to signal commitment and achieve sustained integration of the program across the district.
5. Expand roles of support staff currently supporting SEE Learning, so that they have dedicated time to support the fostering of program growth.
6. Integrate trainings, such as SEE 101, into leadership onboarding.
7. Encourage administrators to be implementors and advocates for the program and for adult SEL.
8. Provide time for presentations, short trainings, and department head involvement to discuss and promote the use of programs such as SEE Learning.
9. Leaders should consider and explore how to provide various learning options that accommodate different needs within the classroom and within special education.
10. Involve upper administration, instructional coaches, and staff in leadership trainings to maximizing program impact.

#### 5.1.2 Restoration

Globally, within educational environments it is crucial to recognize systemic inequities that can be perpetuated (45, 46), and how those inequities can give rise to societal disparities related to race, gender, ethnicity, social class, and other factors (47). To address these concerns, there has been an increasing emphasis on restorative practices being used in schools to provide “safer and more positive school communities, offer alternatives to exclusionary practice, and promote equity” (48). Schools and school districts bear the responsibility of addressing educational equity. Some schools that have adopted SEE Learning as a program have taken specific measures to promote equity. They have done this by emphasizing restoration through culturally responsive classrooms and implementing Positive Behavioral Interventions and Supports (PBIS), among other strategies. These schools have also explicitly included language in their mission or priority statements that addresses historical inequities. By doing so, they have established a clear commitment to incorporating these practices as a fundamental part of their educational approach.

SEE Learning, as a program, plays a crucial role in equipping schools to engage with this work more deeply. It does so by providing the skills and supports necessary to foster compassionate and trauma-informed engagement, both with oneself and with others. As schools undertake the challenging task of navigating and addressing historical inequities, SEE Learning offers a framework that encourages a more profound and holistic approach engaging with compassion. Differential exposure to inequities, especially across generations, is important to consider when addressing social development, health, and wellbeing of all within the community. Neglecting these social injustices, even with the best of intentions, can unintentionally reinforce a kind of “blindness” – whether it’s color blindness, gender blindness, or ability blindness. Such blindness hinders a nuanced understanding of the social environment in which students are embedded on their journey towards self-discovery. As school communities consider integrating restorative practices into their overall approach, skills taught through SEL programming (e.g., self-awareness, compassion for self and others) can serve as an integral part of the implementation process. Further, by combining these approaches, restorative practices can be a means to foster students’ SEL skills, including through communication, kindness, empathy, and care.

To effectively incorporate restorative approaches, schools and districts need to establish clear alignment between these practices and other initiatives including SEL and have a clear and effective communication of this alignment to school communities, including staff, teachers, students, and parents. By engaging an alignment and communication strategy, schools can deepen the incorporation of restorative and culturally responsive practices (49–54).

##### 5.1.2.1 Example of restoration – district alignment

Example: SEE Learning has been aligned with culturally responsive practices within districts to harness intrapersonal development and systemic recognition practices to promote whole community wellbeing. By establishing clear alignments and effectively communicating this alignment to school communities, staff, teachers, and students, schools can gain a deeper understanding of how to incorporate programs like SEE Learning into the curriculum and behavioral management approaches with culturally responsive practices that support authenticity and restoration. This integration aims to promote the seamless inclusion of educational initiatives in daily operations, fostering psychological, academic, and relational growth.

Key actions to promote this alignment in schools implementing SEE learning

1. Articulate how SEE Learning skills and practices supports the district’s strategic plan, including restorative models and broader educational goals, emphasizing the integration of compassion-based practices to support individuals and groups as they navigate restorative and mission objectives.
2. Establish transparent communication channels to inform and engage the entire school community in understanding these alignments.
3. Collaborate with school engagement coordinators and parent program coordinators to promote awareness of SEE Learning and its role as a toolkit to support the community as they navigate achieving educational and mission goals.
4. Communicate the alignment of SEE Learning with the district’s mission to departments and school leadership, underlining the significance of SEE as a supportive tool kit to build compassion-based skills and practices.
5. Integrate SEE Learning practices into the PBIS and other systems of positive behavioral supports to make it an integral part of the educational experience, committed to creating a safe, supportive, and equitable learning environment for teachers, students, and staff.
6. Develop a model school for SEE Learning whole-school implementation that includes alignment initiatives and the ways SEE Learning acts as a toolkit, and using the school as a blueprint for district-wide alignment efforts at all levels.
7. Integrate SEE Learning into the Multi-Tiered System of Support (MTSS) and leverage SEE Learning studies to enhance the effectiveness of support services.
8. Assign dedicated individuals to facilitate the articulation and communication of alignment between restorative and culturally responsive initiatives to support implementation, optimizing the effective use of these tools while managing workloads across the staff.

#### 5.1.3 Integration

Incorporating SEL programs geared towards intrapersonal skills necessitates a systemic approach to implementation (55, 56). This holistic integration underscores the seamless connection between intrapersonal development, interpersonal cultivation, and system-level change within the broader educational landscape. Moreover, it’s crucial to encourage educators to establish resilient peer networks and collaborate with one another, sharing best practices and refining their skills collectively. This collaborative approach extends to the entire educational ecosystem and highlights the profound connection between intrapersonal development, interpersonal skills, and systemic changes in the broader educational landscape.

Integrating SEL programs into the school community is not merely a checklist of activities but a deliberate and holistic transformation that touches upon every facet of the educational system. It requires a commitment to fostering a supportive environment, allocating time, and empowering educators with the tools needed to nurture students’ social and emotional growth.

##### 5.1.3.1 Example of integration – holistic implementation

Example: Schools working with SEE Learning have engaged this by first establishing an implementation strategy for that works for their school. Key to this domain is fostering buy-in by teachers and including teachers in the co-creation of the implementation process, so that the activities integrate into their workload rather than becoming additional demands. In the case of SEE Learning, certified facilitators work with the school to train teachers in the implementation of the program by providing comprehensive teacher training to ensure educators are well-prepared. They also work with schools to provide regular observation and feedback during the initial phases of implementation. The structure of implementation is determined though planning with the trained facilitators. This can occur through classroom delivery and integration into daily activities or cross-curricular integration, incorporating concepts into subject matter.

Key takeaways from schools

1. Establish dedicated spaces and times within the classroom and schools to create a conducive learning environment for SEE Learning integration.
2. Assign dedicated certified SEE Learning facilitators who play a vital role in delivering the program effectively.
3. Regularly observe SEE classes and provide supportive, formative feedback to improve program quality.
4. Work with certified facilitators to design strategies to integrate SEE Learning into various spaces within the school, making it relevant to students and adults alike.
5. Incorporate modern and relevant examples from students’ lives to emphasize and reinforce the practices and skills within the SEE Learning experiences.
6. Ensure communication and collaboration between teachers and with facilitators to help teachers understand and integrate SEE Learning into the classroom and various subjects.
7. Utilize digital systems that may exist within the school or school district, along with digital support materials provided by SEE Learning to facilitate the implementation process and support educators.
8. Create processes that promote buy-in and co-creation by reducing the demands on teachers though structures of learning and support, and by providing teachers with time to learn and practice with the program during their initial exposure to SEE Learning.

#### 5.1.4 Connection and community

The domain of connection and community revolves around the cultivation of genuine relationships and authentic bonds (57) between students, staff, and parents. The objective is to foster an environment where authenticity is nurtured through the entire school community (58). This means infusing the curriculum with narratives and voices representative of the school community and families, providing school communities with opportunities to practice and develop the language and skills being taught in the programs, and infusing community engagement activities with practices and language that reinforce their impact (59).

##### 5.1.4.1 Example of connection and community – community engagement.

Example: in addition to involving all the members of the school community, schools implementing SEE Learning have developed strategies for involving families and community members that may not traditionally be engaged in the business of schooling. Consistent with the Ecological Systems Theory (40), finding ways to incorporate learning into wider systems can lead to more opportunities for modeling and for practice. As such, partnership with families can be vital to supporting SEL concepts and practices. Further, given that the larger community can serve myriad roles including as care providers for students, incorporating them into the learning process can be beneficial as well. Broadening communication channels and actively involving parents and the community in co-creation aligns their values with school priorities, encouraging further engagement. Further, supporting SEE Learning capacity development in the larger community affords more opportunities for teachers to be in conversation with parents about these skills and capacities. This integration underscores the essential notion of authenticity permeating the school community, where narratives, voices, and shared language play an integral role in shaping the educational landscape.

1. Engage all school staff and students in SEE Learning as both learners and leaders, making it an integral part of the school community.
2. Utilize parent meeting spaces to introduce SEE Learning concepts and practices.
3. Share asynchronous resources through platforms like WhatsApp.
4. Expand communication channels to involve a larger parent and community audience.
5. Organize community engagement through knowledge reception, engaged learning, and practice the embodiment stages.
6. Engage teachers and staff in community participation, including authoring lectures, book clubs, newsletter insights, and showcasing student work.
7. Establish community spaces for facilitated conversations with parents.
8. Promote parents and students practicing skills together and offer SEE Learning skills classes for parents.

#### 5.1.5 Educator support

Creating a culture of continuous improvement and learning for adults needs to include acknowledgment of and support with social and emotional competency development among teachers and staff. The Prosocial School Classroom Model has posited that caring for teachers’ wellbeing and social and emotional competence is critical for supporting student social and emotional learning needs (44). Research has demonstrated support for this model, providing evidence for the value of adult social and emotional wellbeing for emotional support in the classroom and for student outcomes (60, 61).

Elevating intrapersonal development through SEL programs hinges on empowering educators through targeted professional development (62, 63). This professional development should be aimed at teaching new pedagogical techniques for working with students and should also support necessary skills for teachers to practice and personally embody the skills which they plan to teach students. Many programs have been designed to support the skills that educators need to foster compassionate and equitable classroom settings (64).

Further, the paradigm shifts towards educator support entails creating communities of practice that provide ongoing guidance and a space for experimentation (65). It underscores the recognition that educators need not be fully adept before implementing change; rather, they can concurrently learn and apply new strategies to cultivate both student and educator mastery (66) while also cultivating their own personal practice (42, 67–74).

Practically, educators need to be given the space to learn how to incorporate SEL into their teaching methods and curricula. It also means supporting their self-efficacy and skills in guiding students to develop intrapersonal skills that are fundamental to the overall wellbeing and academic success of everyone. Finally, this includes supporting the development of nonevaluative spaces that are emotionally safe, learning-focused, and allowing for personal development.

##### 5.1.5.1 Example of educator support – adult SEL & communities of practice

In practice, creating space for educator training can be difficult. Among the schools involved in this project, multifaceted implementation included a formal offering of Cognitively Based Compassion Training (CBCT); CBCT is a professional development program for educators designed to help them hone their own intra- and inter-personal compassion based SEL skills. Planning teams have articulated a range of needs, encompassing guided SEL practices as well as more comprehensive skill-building opportunities, both for personal development and classroom implementation growth. The call for courageous conversations and mindful dialogues is aimed at facilitating sincere discussions surrounding sensitive or challenging subjects. Courageous conversations involve open and honest dialogues that tackle these tough topics, necessitating participants to demonstrate courage through expressing their truths, active listening, and engaging with empathy and vulnerability. Mindful dialogue signifies a form of communication where participants engage in conversations with heightened awareness, presence, and intentionality. This approach is derived from the mindfulness field and is an integral part of the SEE Learning curriculum. Teachers have emphasized the importance of integrating opportunities to extend their own SEL into various school activities, incorporating these skills and practices. This integration is pivotal in fostering intrapersonal and peer-to-peer interaction skills that contribute to a compassionate culture, ultimately enhancing the overall SEL environment within the school.

For example, the schools that we worked with have worked to tailor support, guided by experienced CBCT facilitators, to ensure a customized approach that caters to the unique needs of the adult learning community at the school. This comprehensive strategy fosters a supportive and collaborative school environment, ultimately enhancing the overall educational experience by empowering both educators and students alike.

Further, among the schools we have worked with, the development of communities of practice have been prioritized to support teacher and staff leadership development, adult SEL, and the implementation of SEE Learning in the classroom. Ongoing professional development is important for the long-term sustainability of their SEE Learning initiatives. These communities of practice facilitate collaboration and knowledge sharing among educators and foster a culture of continuous learning and improvement. SEE Learning’s strategies, such as employing certified teachers, conducting regular debrief and support sessions, and forming SEE professional learning communities with team leads, are examples of how communities of practice are being used in application. SEE Learning’s practical implementation of communities of practice demonstrates the viability of this approach for fostering educator support and enhancing intrapersonal development within the educational ecosystem by creating structures for ongoing support.

Key takeaways for educator support:

1. Allocate dedicated time and resources for training to ensure teachers are well-prepared for SEE Learning implementation.
2. Develop a regular structure of training integrated into teacher onboarding activities.
3. Implement CBCT and additional personalized professional development for educators to enhance adult SEL.
4. Provide dedicated space and time for staff to debrief and reflect.
5. Model practices when opportunities arise to encourage participation.
6. Offer small, short sessions for personal skill-building (15 min). For example, incorporate guided SEL practices during staff and morning meetings or host lunchtime drop-ins with small groups to introduce SEE Learning concepts.
7. Create self-care spaces for teachers to prioritize wellbeing.
8. Foster a culture of peer recognition and offer internal development support, while identifying staff leaders to drive sustainability.
9. Build space and time for communities of practice to support ongoing professional development.
10. Utilize trained SEE Learning facilitators, who are trained to run trainings for educators, or experienced teachers to support sessions, and peer workshops.
11. Consider how teacher leaders play a vital role in providing guidance and supervision as a part of the implementation process.
12. Support training in effective communication and co-facilitation for program success.
13. Provide opportunities for formal and informal communities of practice to develop a culture of peer support for teacher, counselors, and staff.

#### 5.1.6 Strength-based cultivation and student voice

Student voice is crucial when reimagining a sustainable approach to whole-school health and wellbeing promotion because students are at developmental periods where they are often more influenced by peer interaction then by adult interactions. Student voice means fostering student leadership in the integration of health promotion into the school community. Developmentally, peer-peer interaction is a critical focus for students. Student leaders as facilitators in the promotion of social and emotional development naturally integrates student voice into the decision-making process, training students in practice, and training students in the facilitation of peer-peer support (75). Taking this approach creates a culture of engagement that further supports sustainability within the school (76).

Further, a strengths-based pedagogical approach fundamentally centers on students, empowering them to recognize their skills and align them with their learning needs. By shifting focus from deficits to strengths, educators lay the groundwork for a flexible mentality that values growth edges. The practical manifestation of this approach involves affording students choices (58), respecting their diversity, nurturing autonomy, cultivating agency, and integrating policies and practices anchored in this strengths-based philosophy (77). Additionally, while a strength-based pedagogical orientation centers on students, it is also possible to extend a strengths-based approach to adult interactions to create a sense of mastery that can ground the school community in a strengths-based orientation towards whole-school growth and development.

##### 5.1.6.1 Example of student voice and strengths-based cultivation – student voice

Example: SEE Learning places significant emphasis on incorporating student voice and empowerment as essential components of leadership development within SEL. Schools implementing this program have worked to identify ways of integration student voice into the planning, training, and ongoing development of SEL to foster a culture of engagement that enhances SEL sustainability. SEE Learning’s model demonstrates how integrating student voice and leadership strengthens the school community and supports SEL sustainability by empowering students to recognize their skills, respect diversity, and nurture their autonomy and agency. This approach fundamentally aligns with a strengths-based pedagogy, valuing student growth edges and fostering a flexible mentality that centers on students’ strengths and needs.

Key takeaways

1. Affinity clubs and groups with student leaders create spaces for student engagement.
2. Integrating student-led practices into The Student Government Association (SGA) provides students with leadership opportunities.
3. Peer leadership programs can provide opportunities for older students to support younger students entering the school or transitioning into a new grade level.
4. Practices can be integrated into service leadership boards that engage students in community service initiatives and work to integrate SEE Learning components into service-learning project development.
5. As a community there can be focus on creating, communicating, and building awareness of opportunities for student voice and leadership as they arise.

## 6 Discussion

This paper began with a focus on two distinct paths to success: the Angelou Path and the Icarus Path. The Angelou Path champions success through perseverance, resilience, and positive relationships, ultimately leading to personal growth and the ability to withstand challenges. In contrast, the Icarus Path is marked by a reckless individual pursuit of achievement, often at the cost of wisdom and long-term consequences, ultimately harming the individual and overlooking personal and community development. To reorient success as supportive of the whole child and, ultimately, the whole community, a fundamental shift in the educational system is required. This shift can be achieved by embracing the PRICES model, which serves as a framework for integrating SEL programs into the educational landscape as health and wellbeing initiatives. The framework emphasizes the importance of looking beyond the individual and considering the dynamics of relationships among individuals and the broader system, including policies and practices, when striving for sustained change.

Maya Angelou’s emphasis on fostering connection and belonging aligns seamlessly with the pivotal role of schools in nurturing social and emotional development. While schools may produce high-achieving students without such a focus, adopting an Icarus-like approach, prioritizing individual achievement while neglecting communal support and holistic wellbeing, could lead to eventual setbacks. Therefore, it is essential to redefine schools as environments that prioritize enduring outcomes, cultivate a sense of community, interconnectedness, and belonging, and focus on the holistic wellbeing of students and educators. This redefinition allows educational institutions to significantly influence the health and wellbeing of their communities.

Acknowledging schools as potent agents of health promotion necessitates the implementation of evidence-based interventions and comprehensive support to navigate challenges. The integration of SEL within the educational fabric emerges as a critical intervention that educators and educational leaders should prioritize. Such integration has the potential to positively impact the mental health and overall wellbeing of school-age children, ultimately fostering their flourishing.

In the story of Icarus, unchecked ambition led to a magnificent accomplishment but ultimately cost him his future. Similarly, reimagining schools rooted in the PRICES model fosters the capacity of individuals and communities to rise above adversity, sustained not solely by solitary achievement, but by the collective strength of relationships and shared experiences. The enduring wisdom of Angelou’s collective resilience and the cautionary tale of Icarus illuminate a path forward—a path where schools become nurturing grounds not only for academic prowess but also for the holistic development and thriving of each student.

## Data availability statement

The original contributions presented in the study are included in the article/supplementary materials, further inquiries can be directed to the corresponding author.

## Author contributions

TF: Conceptualization, Writing – original draft, Writing – review & editing. SD: Writing – review & editing.
